# Association of Periodontitis and Atopic Dermatitis with the Levels of IL-13, IL-31, and TSLP in the Gingival Crevicular Fluid

**DOI:** 10.3390/ijms242115592

**Published:** 2023-10-26

**Authors:** Constanza Jiménez, Javier Fernández, Marcela Aroca, María José Bordagaray, Elizabeth Pellegrini, Javier Contador, Marcela Hernández, Fernando Valenzuela, Alejandra Fernández

**Affiliations:** 1 Faculty of Dentistry, Universidad Andres Bello, Echaurren 237, Santiago 8370133, Chile; c.jimenezlizama@uandresbello.edu (C.J.); ma.chandia92@gmail.com (M.A.); 2 International Center for Clinical Studies (CIEC), Probity Medical Research, Manzano 343, Santiago 8420383, Chile; fernandez.moraga.javier@gmail.com; 3 Laboratory of Periodontal Biology, Faculty of Dentistry, Universidad de Chile, Olivos 943, Santiago 8380544, Chile; mbordagaray@odontologia.uchile.cl (M.J.B.); elizabeth.pellegrini95@gmail.com (E.P.); mhernandezrios@odontologia.uchile.cl (M.H.); 4 Department of Dermatology, Faculty of Medicine, Universidad de Los Andes, Av. Plaza 2501, Santiago 7620157, Chile; jicontador@uc.cl; 5 Department of Pathology and Oral Medicine, Faculty of Dentistry, Universidad de Chile, 943 Olivos Street, Santiago 8380544, Chile

**Keywords:** atopic dermatitis, periodontitis, gingival crevicular fluid, interleukins, interleukin-13, interleukin-31, thymic stromal lymphopoietin, biomarkers

## Abstract

Emerging epidemiological evidence links atopic dermatitis (AD) and periodontitis, although the mechanisms remain unclear. Th2-derived cytokines are key in the development of both diseases, and different gingival crevicular fluid (GCF) profiles among healthy and diseased subjects have been previously reported. This case–control study examined the GCF levels of interleukins (IL)-13, IL-31, and thymic stromal lymphopoietin (TSLP) in 29 subjects with moderate-to-severe AD and 33 controls. All subjects underwent comprehensive clinical and oral evaluations, followed by GCF collection. GCF levels of IL-13, IL-31, and TSLP were assessed using a multiplex-bead immunoassay. Demographic and periodontal parameters were similar among groups (*p* > 0.05). The GCF levels of IL-31 and TSLP were higher in AD subjects compared to controls (*p* < 0.05), whereas no significant differences in the GCF levels of IL-13 were noticed (*p* = 0.377). Moderate-to-severe AD was positively associated with the GCF levels of IL-31 and TSLP, whereas severe periodontitis was negatively associated with IL-31 (*p* < 0.05). The GCF levels of IL-13 showed no significant associations with either condition (*p* = 0.689). There was no significant interaction between AD and periodontitis for IL-31 (*p* < 0.869). These results suggest that AD and periodontitis independently influence the GCF levels of IL-31 in opposing ways, whereas AD alone influences the levels of TSLP.

## 1. Introduction

Periodontitis is a chronic inflammatory disease that compromises the integrity and function of dental support structures, including the gingiva, periodontal ligament, and alveolar bone. It is particularly prevalent in adults, with an estimated 1.1 billion people worldwide suffering from severe forms of the disease [1]. Although infection is necessary for the onset of periodontitis, it alone cannot drive its progression. Instead, factors such as individual susceptibility and dysregulation of the Th1, Th2, Th17, and Th-reg inflammatory responses play a crucial role in its progression [2]. Research indicates that in the presence of an antigenic stimulus, patients with progressive periodontitis have predominantly activated Th2 lymphocytes [3,4].

Like periodontitis, the pathogenesis of atopic dermatitis (AD) is partially explained by the imbalance of Th1, Th2, Th17/23, and Th22 subsets in response to epithelial microbial dysbiosis [5,6,7,8]. AD is primarily mediated by the Th2 immune response within the skin, which plays a key role in the development of the disease’s characteristic cutaneous inflammation [9]. This Th2-dominated immune response relies on the thymic stromal lymphopoietin (TSLP) signaling pathway [10]. TSLP is a cytokine secreted by skin keratinocytes that enhances the proliferation of Th2 cells and the successive production of IL-13 and IL-31 [11,12,13,14].

IL-13 and IL-31 are Th2-cell-derived cytokines identified to play significant roles in the symptoms and pathogenesis of AD [15]. In this regard, animal studies have demonstrated that intradermal injections of IL-13 serve as a direct pruritogen in mice models of the disease, while intradermal injections of IL-31 function as an indirect pruritogen by means of increasing the production of leukotriene B4 in keratinocytes [16,17]. Clinical research shows a significant increase in the expression levels of TSLP and IL-31 in the serum of AD patients compared to controls [18]. Likewise, the serum levels of IL-13 mRNA were also significantly overexpressed in AD subjects versus non-AD individuals [19]. In addition, cultivated peripheral mononuclear cells from AD patients have been reported to overexpress IL-13 in vitro [20]. These findings, coupled with AD’s associations with other disorders such as cardiovascular disease [21], strongly reinforce the notion that AD is not merely a “localized skin disease” but an actual systemic disorder capable of affecting distant organs [22]. Within this framework, both IL-13 and IL-31 may be central biological mechanisms involved in these associations.

Few studies have explored the association between AD and periodontal diseases [23,24]. Although AD and periodontitis share etiopathogenetic mechanisms and comorbidities, the existing literature does not provide enough evidence to ascertain whether AD can influence the immunoinflammatory response of periodontal tissues [25]. In this context, a recent publication successfully associated AD with gingival inflammation and periodontitis (adjusted OR = 1.69, 95% CI: 1.38 to 2.08 and 1.42, 95% CI: 1.13 to 1.77, respectively) [23], whereas a second study reported that AD influences the gingival crevicular fluid levels of MMP-8 independently of periodontal status [25]. In addition, a positive link between AD and caries has also been reported in adults [23,26], and the disease has been shown to cause inflammatory changes in the salivary glands of animal models [27]. These findings suggest that AD may be involved in inflammatory changes in the oral cavity.

Gingival crevicular fluid (GCF) is a serum transudate constantly produced and secreted into the gingival crevice surrounding teeth. Its main function is to purge foreign bacteria from the crevice, acting as a natural barrier against microbial invasion [28]. A study suggested that molecules from systemic diseases can travel to the oral gingiva, modifying the immune composition of the GCF [29]. Prior studies using multiplex-bead immunoassays and ELISA methods have uncovered distinct GCF profiles between systemically healthy subjects and those with acute myocardial infarction, diabetes, rheumatoid arthritis, and psoriasis [29,30,31,32,33,34]. However, this aspect has yet to be comprehensively evaluated in AD.

Since the Th2 immune response plays a crucial role in the pathogenesis of both periodontitis and AD, and these diseases can modify inflammatory cytokines at a systemic level, we hypothesize that AD and periodontitis are responsible for modulating the GCF levels of key Th2-cell-derived cytokines. Therefore, this study aims to compare the GCF levels of IL-13, IL-31, and TSLP between AD patients and healthy controls and to explore the impact of periodontitis and AD on the GCF levels of these molecules.

## 2. Results

GCF samples were obtained from a cohort of 62 consenting and volunteering adults, including 29 patients with moderate-to-severe AD and 33 dermatologically healthy controls (C). Demographic and clinical periodontal parameters are summarized in Table 1. Statistical analysis revealed no significant intergroup differences across any of the measured variables; however, individuals with moderate-to-severe AD presented a non-significant yet slightly higher frequency of severe periodontitis (*p* = 0.199).

**Figure 1:**
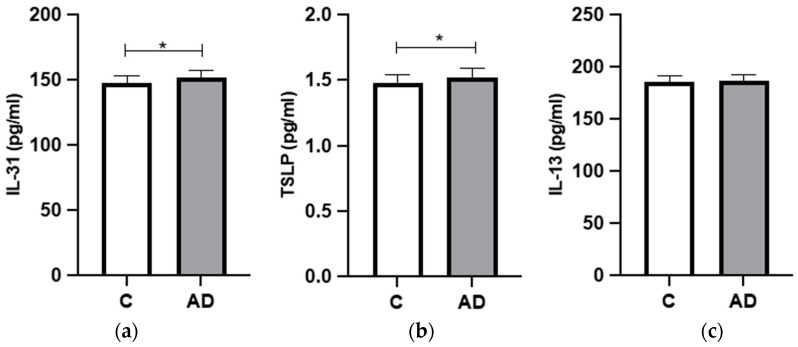
GCF biomarker levels in AD patients and healthy controls. (**a**) Interleukin-31 levels, (**b**) TSLP levels, and (**c**) IL-13 levels. IL, interleukin. TSLP, thymic stromal lymphopoietin protein. C, dermatologically healthy controls. AD, atopic dermatitis; *, *p*-value < 0.05 at a 95% confidence interval. *p*-values were calculated using Student’s *t*-test.

The GCF levels of the researched biomarkers are depicted in Figure 1 and Table 2. The GCF levels of interleukin (IL)-31 and thymic stromal lymphopoietin (TSLP) proteins were significantly higher in moderate-to-severe AD patients compared to controls (*p* < 0.05). The GCF concentrations of IL-13, on the other hand, showed no statistically significant differences among groups (*p* = 0.377).

A multiple regression analysis was performed to evaluate the impact of AD diagnosis and the covariates age, gender, smoking status, and periodontitis severity on the GCF levels of IL-13, IL-31, and TSLP. The results are summarized in Table 3. The covariates age, gender, and smoking status yielded no significant impact on the GCF levels of the studied molecules. However, a significant positive association was observed between moderate-to-severe AD diagnosis and the GCF levels of IL-31 and TSLP (*p* < 0.05, respectively). In contrast, a significant negative association was found between severe periodontitis diagnosis and the GCF levels of IL-31 (*p* < 0.05). The combined effects of moderate-to-severe AD and severe periodontitis accounted for 16.5% of the variability in the GCF levels of IL-31, as determined by the corrected goodness-of-fit measure for linear models (Adj. R^2^ = 0.165). Similarly, moderate-to-severe AD explained 2% of the variability in the GCF levels of TSLP (Adj. R^2^ = 0.023). Notably, the GCF levels of IL-13 did not show significant associations with any of the researched variables (*p* = 0.689). Therefore, these results were not included in the table.

**Figure 2:**
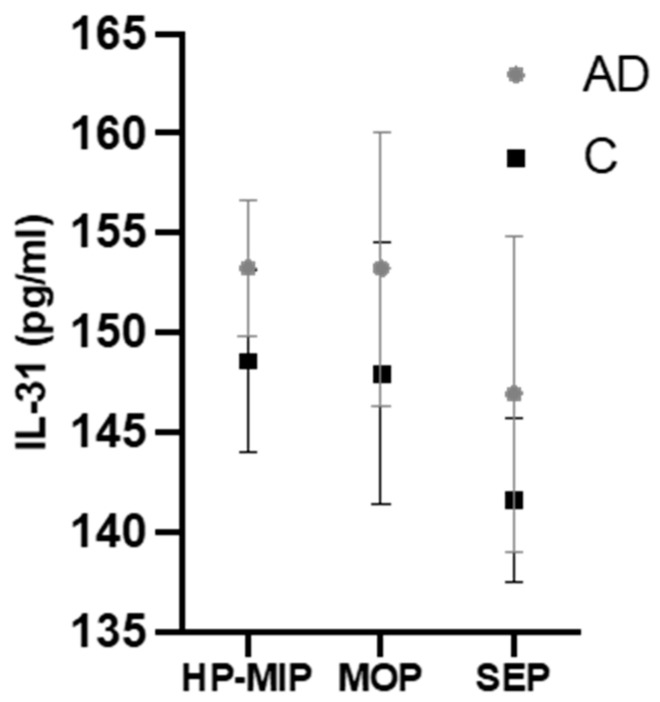
Mean GCF levels in AD patients and healthy controls according to periodontitis severity. AD, atopic dermatitis; C, dermatologically healthy controls; HP, healthy periodontium; MIP, mild periodontitis; MOP, moderate periodontitis; SEP, severe periodontitis.

Finally, a two-way analysis of variance (ANOVA) was performed on a sample of 58 patients to explore further the effects of moderate-to-severe AD diagnosis and severe periodontitis on the GCF levels of IL-31. The results are shown in Table 4. The test found no significant interaction between both variables and the GCF levels of IL-31 (*p* < 0.05). Briefly, moderate-to-severe AD patients consistently presented higher GCF levels of IL-31 than healthy controls, regardless of periodontal status and periodontitis severity (Figure 2). Mean levels of GCF IL-31 in AD patients with no/mild, moderate, and severe periodontitis were 153.273 ± 3.424, 153.233 ± 6.873, and 146.951 ± 7.931 pg/mL, respectively. Mean levels in the controls were 148.579 ± 4.522, 147.964 ± 6.542, and 141.647 ± 4.093 pg/mL, respectively.

## 3. Discussion

AD is an inflammatory skin condition sustained by genetic mutations, environmental factors, and an altered immune status. Previous studies have demonstrated that GCF is a suitable and non-invasive sampling procedure that shows different profiles in individuals with chronic pathologies compared with controls [29,30,31,32,33,34], while the GCF profile in individuals with AD remains elusive. In our study, we determined for the first time that AD individuals have higher levels of IL-31 and TSLP in GCF than controls. In addition, we also showed that AD and periodontitis influence IL-31 in opposing and independent ways, while TSLP levels are only influenced by AD diagnosis.

IL-31 is a key cytokine involved in the clinical manifestations of AD, being particularly important in the development of the severe pruritus that characterizes the disease [35,36,37]. In relation to that, studies have found that the expression levels of IL-31 are significantly overexpressed in itchy AD skin samples from AD patients compared to healthy controls, reinforcing the local role of IL-31 in promoting pruritus [38]. On a systemic level, a recent meta-analysis revealed that the serum concentrations of IL-31 are also higher in AD patients compared to healthy controls [37], suggesting that AD presents extra-skin consequences that have been far less studied.

In this study, we evaluated for the first time whether the levels of IL-31 in oral fluids, specifically gingival crevicular fluid, could be modified in AD patients compared to healthy controls. As we anticipated, we found that individuals with AD showed an upregulation of IL-31 in the GCF compared to healthy controls. These outcomes suggest that AD might cause changes in the IL-31 concentrations in periodontal tissues. While the role of IL-31 in periodontal tissues is still not fully elucidated, the topic of study is rapidly evolving and developing [39]. An in vitro study showed that infection with *Porphyrormonas* (*P*.) *gingivalis* increased the expression of the IL-31 receptor (IL-31R) in human gingival epithelial cells and that IL-31 in those cells downregulates the *P. gingivalis*-induced overexpression of claudin-1 [40]. Since claudin-1 regulates epithelial homeostasis [41], it is feasible to theorize that in the presence of *P. gingivalis*, IL-31 favors the development of gingival epithelial barrier dysfunction, potentially compromising the periodontal defense. Clinical evidence shows higher levels of IL-31 in both the GCF and saliva of periodontitis patients compared to their healthy counterparts and that those levels were seen to decrease after periodontal treatment, implying a potential role of IL-31 in the pathogenesis of periodontitis [39,42].

IL-31 is a Th-2 cell-derived cytokine that modulates the pro-inflammatory responses in immune cells, intestinal epithelial cells, and colonic subepithelial myofibroblasts [43,44,45]. In monocytes and macrophages pre-stimulated with staphylococcus exotoxins, IL-31 induces the secretion of IL-1 and IL-6 and upregulates the secretion of IL-18 [45]. These pro-inflammatory cytokines are implicated in osteoclast differentiation [46,47,48], an essential process in the pathogenesis of periodontitis [49]. Thus, in the presence of oral exotoxins, it is plausible that IL-31 orchestrates the inflammatory response within periodontal tissues. In addition, IL-31 is likely to have an indirect role in the extracellular matrix remodeling of periodontal tissues, given its ability to induce the secretion of matrix metalloproteinases (specifically, MMP-1, MMP-3, MMP-25, and MMP-7) in human colonic subepithelial myofibroblasts [43]. Therefore, investigating the potential periodontal implications of higher IL-31 levels in the GCF should be a valuable direction for future studies.

While periodontitis and AD share similar inflammatory pathways, our study shows that both diseases influence the GCF levels of IL-31 in opposite and independent ways: AD was positively associated with the GCF levels of IL-31, whereas severe periodontitis was negatively associated with the GCF levels of the interleukin. In this regard, we theorize that moderate to severe AD increases the expression of IL-31 in the skin, and that this overexpression allows for the translocation of IL-31 into the systemic circulation due to the edema and hyperpermeability of local blood vessels. This phenomenon may be partly attributed to the known functions of vascular endothelial growth factor (VEGF) molecules in AD [50]. Consequently, enhanced levels of IL-31 overpass into the periodontal tissues from the systemic circulation, a process which may be further facilitated by the vascular changes inherent to periodontitis.

Recently, it has been suggested that inflammatory bowel disease affects the expression levels of cytokines in the gingival tissues [51,52]. Contrary to our results, it appears that inflammatory bowel disease may negatively influence the levels of IL-31 in the gingival tissues [51]. On the other hand, severe periodontitis can also negatively affect the levels of IL-31 in the GCF. It is known that *P. gingivalis*, through its lysine-specific protease gingipain, induces the secretion of IL-31 in human mast cells via the JNK and NF-κB signaling pathways [40]. In addition, a lower number of mast cells has been reported in the periodontal mucosa of patients with severe periodontitis compared to those with moderate periodontitis [53]. Therefore, it is plausible that the GCF levels of IL-31 in periodontal tissues depended on the mast cell count relative to the severity of periodontitis.

Protease allergens and microorganisms can stimulate the secretion of TSLP from epithelial and immune cells [54]. TSLP belongs to the IL-2 cytokine family and participates in physiological and pathological conditions. On the one hand, TSLP regulates dendritic cells (DCs) to guide the development of regulatory T cells (Treg). On the other hand, TSLP can also promote Th2 responses [54].

TSLP is known to be overexpressed in the keratinocytes of AD patients, in stark contrast to healthy controls where the cytokine is undetectable [55]. Further studies reinforce these findings, reporting that both the gene and protein levels of TSLP are significantly upregulated in skin samples from AD subjects compared to healthy controls [18,56,57]. As theorized, our study found higher levels of TSLP in the GCF of AD patients compared to healthy controls [58]. Furthermore, a detailed multiple regression analysis proved that the observed upregulation was specifically attributable to AD and not periodontitis [58]. These findings lead us to believe that TSLP, which is highly overexpressed in the skin of AD subjects, can enter the systemic circulation and subsequently translocate to the periodontal tissues. However, additional research is still needed to validate this theory.

The role of TSLP in periodontal tissues remains unclear; nonetheless, it is possible that the cytokine participates in periodontitis progression [59]. Supporting this idea, in vitro studies have found that stimulation with *Aggregatibacter* (*A.*) *actinomycetemcomitans*, a key periodontal pathogen, leads to an increase in the TSLP mRNA expression in epithelial cells and macrophages as compared to unstimulated cells [59]. Moreover, a study reported a detection frequency of 5.9% for TSLP in inflamed periodontal sites of patients with gingivitis and 9.1% in inflamed periodontal sites of patients with periodontitis. The cytokine was not detected in non-inflamed periodontal sites of periodontally healthy subjects and non-inflamed sites of patients with gingivitis or periodontitis [60].

Animal models demonstrate that IL-31, IL-13, and IL-4 are essential molecules involved in the imbalance and dysfunction of the skin barrier in AD. These cytokines hinder keratinocyte differentiation and reduce the expression of tight junction protein claudin (Cldn)-1 in the lower layers of the epidermis [61]. Clinically, studies show that patients with AD display higher levels of IL-13 gene expression in the blood as compared to individuals without the disease. Moreover, an association between IL-31 gene expression and AD severity has also been reported, with severe AD subjects displaying higher IL-31 gene expression levels than those with mild or moderate forms of the disease [19]. Conversely, our research did not find any differences in the levels of GCF IL-13 in patients with moderate/severe AD and healthy controls. Therefore, we believe that AD does not impact the immune response related to IL-13 in periodontal tissues, or that this cytokine, which is expected to be upregulated in the blood of moderate and severe AD, does not reach the GCF.

### Limitations

The results of this research should be interpreted with caution due to inherent design-related biases. Like other observational and retrospective studies, the present work presents limitations in establishing causal relationships and temporal sequencing between the exposures and outcomes. Additionally, there may be unaccounted-for confounding variables that could impact the observed associations. Finally, the limited sample size restricts the study’s generalizability to broader populations. Further research is, therefore, needed to address these limitations and extend the applicability of our findings.

## 4. Materials and Methods

### 4.1. Study Design

The Scientific and Bioethics Committee of the Faculty of Dentistry at Andrés Bello University (UNAB), Santiago, Chile (no. PROPRGFO_002019_80) reviewed and approved the following research protocol. All subjects provided written informed consent prior to enrollment under the ethical standards established by national and international institutions and the Helsinki Declaration [62]. The final manuscript was prepared following the “STrengthening the Reporting of OBservational studies in Epidemiology” (STROBE) guidelines to ensure the transparent and comprehensive reporting of observational studies [63].

### 4.2. Participants

Volunteers diagnosed with AD and healthy controls (C) were conveniently enrolled between March and December 2018 at the International Center for Clinical Studies (CIEC) in Santiago, Chile, and the Dental Clinic of the Faculty of Dentistry at UNAB in Santiago, Chile, respectively. Eligible subjects met the following inclusion criteria: (i) age over 18 years and (ii) possession of at least twelve functional teeth, excluding third molars [25,64,65,66]. In accordance with the study design, the following exclusion criteria were applied: (i) individuals with systemic or dermatological disorders other than AD, especially those characterized by immunoinflammatory dysregulation (such as diabetes, lupus, psoriasis, and rheumatoid arthritis, among others) [25,64], (ii) individuals who had received antibiotics, anti-inflammatory or immunomodulatory drugs within the past three months, (iii) patients who had undergone chemo- or radiotherapy in the previous twelve months, (iv) pregnant females, and (v) patients who had received treatment for AD or periodontitis in the last three months [25,64].

### 4.3. Physical, Dermatological and Intraoral Examinations

Subjects underwent comprehensive physical, dermatological, and intraoral evaluations conducted by a specialized team of dermatologists. The medical history and sociodemographic characteristics were meticulously recorded in predefined charts. AD was diagnosed by a qualified dermatologist based on the patient’s personal and familial disease history, as well as the presence of skin lesions and pruritus. Individuals displaying signs or symptoms indicative of an undiagnosed disease (e.g., diabetes, hypertension, thyroid disorders, etc.) were excluded from the study and promptly referred by the dermatologist to an appropriate medical physician for further evaluation.

An experienced periodontist conducted intraoral examinations. Full-mouth periodontal charting was performed using a manual periodontal probe (UNC-15, Hu-Friedy, Chicago, IL, USA), documenting the following parameters at six sites per tooth (excluding the third molars): (i) bleeding on probing index (BOP), (ii) periodontal probing depth (PD), and (iii) clinical attachment loss (CAL). Periodontitis was diagnosed based on the joint case definition proposed by the American Academy of Periodontology (AAP) and the Centers for Disease Control and Prevention (CDC) of the United States of America [67]. Severe periodontitis was defined as >2 interproximal sites with CAL > 6 mm (not on the same tooth) and >1 interproximal site with PD > 5 mm. Moderate periodontitis was defined as >2 interproximal sites with CAL > 4 mm (not on the same tooth) or >2 interproximal sites with PD > 5 mm (not on the same tooth). No/mild periodontitis was defined as neither moderate nor severe periodontitis [67]. Finally, all patients diagnosed with periodontitis were referred to the Periodontology Clinic at the Teaching Dental Hospital of the Faculty of Dentistry at UNAB, Santiago, for further evaluation and appropriate treatment.

### 4.4. Gingival Crevicular Fluid (GCF) Sampling

As previously described, GCF samples were collected by a trained periodontist from the deepest site per quadrant [25,68]. To prevent saliva contamination, selected sites were carefully isolated using sterile cotton rolls and gently dried with an air syringe. Sterile periodontal strips were placed into the gingival crevice for 30 s until minor resistance was felt. Afterward, the strips were transferred into 2 mL sterile tubes and immediately stored at −20 °C for further analysis at the Periodontal Biology Laboratory of the Faculty of Dentistry at the University of Chile, Santiago, Chile.

### 4.5. Gingival Crevicular Fluid (GCF) Determinations

All analyses were conducted by the same team of lab experts, using pooled samples from each individual [24,25]. A specific elution was prepared by adding forty microliters of protein buffer to each tube. The dilutions were then incubated for thirty minutes at 4 °C and centrifuged for five minutes at 12,000× *g*, maintaining the same temperature. The process was repeated twice to ensure optimal protein isolation.

Afterward, samples were analyzed using a commercially available human multiplex bead immunoassay (R&D Systems, Minneapolis, MN, USA) following the manufacturer’s instructions. For this step, samples were diluted to 1:50 using the panel kit buffer provided. Interleukin (IL)-13, IL-31, and thymic stromal lymphopoietin (TSLP) protein were identified and quantified using a digital platform (Magpix^®^ Merck Millipore, Billerica, MA, USA) and the MILLIPLEX AnalystR software v5.1 (Milliplex AnalystR^®^, Viagene Tech, Minneapolis, MN, USA).

### 4.6. Sample Size Calculation

Sample size requirements for this research were determined using previously reported concentrations of IL-13 in the serum of patients with DA and systemically healthy controls [69,70]. Based on an estimated effect size of 1.47, a significance level of α = 0.05, and a power of 0.8, a minimum of 14 patients per group was deemed necessary to achieve an optimal sample size for this study.

### 4.7. Statistical Analysis

All statistical analyses were conducted using the Stata v13 software (StataCorp. LLC, College Station, TX, USA). The dataset was initially assessed for distribution and homoscedasticity using the Shapiro–Wilk and Levene’s tests, respectively. Since the data exhibited a normal distribution, inferential analyses were carried out using Student’s *t*-test and Fisher’s exact tests at a significance level of 0.05. Next, three multiple linear regression models were performed, taking into consideration IL-13, IL-31, and TSLP levels in the gingival crevicular fluid (GCF) as dependent outcome variables, and atopic dermatitis (AD) and periodontitis as independent or explanatory covariables. This analysis aimed to assess the relationships between these variables while controlling for other influential factors (gender, age and smoker status). Finally, a two-way ANOVA was conducted on a sample of 58 patients to examine the effects that moderate-to-severe AD diagnosis and severe periodontitis could have on the GCF levels of IL-31.

## 5. Conclusions

In conclusion, the principal findings of this study indicate that both AD and severe periodontitis independently and opposingly affect the GCF levels of IL-31. At the same time, AD alone impacts the GCF levels of TSLP. A limitation of this research lies in its observational design, which restricts the ability to ascertain causality for the reported associations.

## Figures and Tables

**Table 1:** Demographic and clinical periodontal parameters of enrolled subjects.

Parameters	C (n = 33)	AD (n = 29)	*p*
Age (years, mean ± SD)	37.27 ± 13.43	31.34 ± 12.80	0.091
Gender: Female (freq., % − n)	69.70 − 23	65.38 − 17	0.784
Smoker (freq., % − n)	40.00 − 12	19.23 − 5	0.145
PD (mm, mean ± SD)	2.16 ± 0.54	2.01 ± 0.38	0.245
CAL (mm, mean ± SD)	1.93 ± 1.43	1.96 ± 0.72	0.660
BOP (positive sites, mean freq., %)	0.46	0.06	0.385
No/mild periodontitis (% − n)	48.48 − 16	34.62 − 9	0.199
Moderate periodontitis (% − n)	42.42 − 14	38.46 − 10	
Severe periodontitis (% − n)	9.09 − 3	26.92 − 7	

*p*, *p*-value; AD, atopic dermatitis; SD, standard deviation; n, number; freq., frequency; PD, probing depth; CAL, clinical attachment level; BOP, bleeding on probing index; positive sites, sites that bled upon periodontal examination. *p*-values were calculated using Student’s *t*-test and Fisher’s exact tests.

**Table 2:** GCF biomarker levels in AD patients and healthy controls.

Cytokine	C (n = 33)	AD (n = 29)	*p*
IL-31 (mean ± SD)	147.68 ± 5.64	151.18 ± 6.39	**0.026**
TSLP (mean ± SD)	1.47 ± 0.07	1.51 ± 0.07	**0.040**
IL-13 (mean ± SD)	185.19 ± 6.04	186.55 ± 5.91	0.377

*p*, *p*-value. Bold = *p* < 0.05 at a 95% confidence interval. C, controls; AD, atopic dermatitis; IL, interleukin; TSLP, thymic stromal lymphopoietin protein; SD, standard deviation. Levels expressed in pg/mL. *p*-values were calculated using Student’s *t*-test.

**Table 3:** Multiple regression models for the GCF levels of IL-31 and TSLP.

Variables	IL-31		TSLP	
	Coef. ± SE	*p*	Coef. ± SE	*p*
Moderate/severe AD	4.215 ± 1.755	**0.020**	0.045 ± 0.020	**0.036**
Moderate periodontitis	−0.315 ± 1.804	0.862	−0.020 ± 0.022	0.366
Severe periodontitis	−6.220 ± 2.407	**0.013**	−0.018 ± 0.029	0.547
Constant	150.209 ± 2.791		1.457 ± 0.031	
Prob > F	0.021		0.267	
Adj. R^2^	0.165		0.023	
No. observations	55		58	

*p*, *p*-value. Bold = *p* < 0.05 at a 95% confidence interval. Standard errors are reported as ± values. AD, atopic dermatitis; IL, interleukin; TSLP, thymic stromal lymphopoietin protein; Coef., coefficient; SE, standard error. Adjusted by gender, age and smoker status.

**Table 4:** Impact and interaction of moderate-to-severe AD diagnosis and moderate or severe periodontitis in the GCF levels of IL-31.

Variables	IL-31	
	Coef. ± SE	*p*
AD and moderate periodontitis	0.575 ± 3.458	0.869
AD and severe periodontitis	0.610 ± 4.671	0.896
Constant	148.579 ± 1.449	
Adj. R^2^	0.159	

*p*, *p*-value. Note: *p* < 0.05 at a 95% confidence interval. AD, atopic dermatitis; IL, interleukin; GCF, gingival crevicular fluid; Coef., coefficient; SE, standard error. Two-way ANOVA.

## Data Availability

The data presented in this study are available on request from the corresponding author.
